# Behavioural Pathways Linking Fast-Food Consumption and Obesity Among Adolescents of Urban Puducherry: Evidence From a Structural Equation Modeling Study

**DOI:** 10.34172/jrhs.13908

**Published:** 2026-08-15

**Authors:** Nirupama Kamaraj, Vedapriya Dande Rajasekar, Deena Divakaran Sreelatha, Suganya Ezhilarasan

**Affiliations:** ^1^Department of Community Medicine, Sri Venkateswara Medical College Hospital and Research Institute, Redhills, Chennai, Tamil Nadu, India; ^2^Department of Community Medicine, Chettinad Hospital and Research Institute, Chettinad Academy of Research and Education, Kelambakkam, Tamil Nadu, India

**Keywords:** Adolescent nutrition, Fast foods, Obesity, Behavioural epidemiology, SEM

## Abstract

**Introduction:**

Children often consume fast food and junk food due to limited time, taste preferences, peer pressure, and a lack of awareness regarding their harmful effects. These foods are high in calories, salt, and sugar but lack essential nutrients. This study assessed the prevalence of fast-food consumption and examined behavioural pathways linking knowledge, attitude, and practice (KAP) with obesity among urban Indian adolescents.

**Study Design:**

This study employed a cross-sectional design.

**Methods:**

This study was conducted in selected schools in the urban areas of Puducherry. A total of 455 adolescents aged 15-18 years were selected from the study population. Chi-square test, Pearson’s correlation, receiver operating characteristic (ROC) curve analysis, and structural equation modeling (SEM) were used to analyze the data.

**Results:**

The prevalence of fast-food consumption was 93.9%; 11.1% 11.1% of participants were overweight, and 15% were obese. A significant association was found between practice score and obesity (*P*=0.016). SEM analysis revealed a positive impact of knowledge on attitude (*P*<0.001) and practice (*P*=0.045); furthermore, attitude significantly influenced practice (*P*<0.001). Practice was inversely related to obesity (β=-0.28, *P*=0.003). ROC analysis indicated that the practice score moderately predicted obesity (AUC=0.692; *P*<0.001).

**Conclusion:**

Dietary practice is a key modifiable behavioural determinant of adolescent obesity. Behaviour-focused school health interventions and nutritional screening strategies may improve obesity prevention among adolescents.

## Introduction

 Fast food culture has become a global phenomenon, with a drastic increase in outlets originating in Southern California. Fast food is described as ultra-processed, high in calories, salt, sugar, and saturated fat, often exceeding recommended dietary allowances. Packaged foods such as chips, carbonated soft drinks, ice cream, baked goods, chocolates, and fried foods are commonly classified as junk foods.^1^ The terms “fast food” and “junk food” are often used interchangeably. This shift has been driven by rapid urbanization, lack of time, a westernized diet, and easy availability. According to UNICEF (2012), high fast-food consumption (55-58%) among teenagers is linked to a 2% increase in body fat percentage and a 23% higher risk of obesity. Factors such as age, gender, Body Mass Index (BMI), soft drink intake, physical activity levels, and sedentary behaviour were associated with fast-food consumption. ^2^

 In India, fast-food culture has increased rapidly, growing by 40% annually, resulting in India ranking tenth globally in per capita fast-food spending (2.1%). ^3,4^ The major drivers include exposure to western cuisine, a rise in nuclear families and working parents, which have significantly impacted the growth of the fast-food industry in the country. ^5^ The National Consultative Group of the Indian Academy of Paediatrics (IAP) report on the consumption of fast and junk foods (2019) noted an increase in total energy intake, characterized by higher carbohydrate and saturated fat content in fast food and junk food. ^6^ Experts have reported elevated odds of developing diabetes mellitus and coronary heart disease among individuals with high fast-food and junk-food consumption over a decade.^6,7-14^

 Various studies in India have reported that factors such as palatability, easy accessibility, greater convenience, and affordability drive the preference for and transition toward fast-food dishes. ^8^ Frequent consumption of fast food among children and adolescents is associated with an increased BMI and insulin resistance. ^9,13,14^ Additionally, clinical studies confirm that the nutrient value of these foods is lower than recommended levels, contributing to various adverse health outcomes. ^9,10^ Urbanization, changing family structures, academic pressure, increased disposable income, and widespread availability of commercially prepared foods have collectively reshaped dietary behaviours among Indian adolescents.^15-18^

 The Knowledge–Attitude–Practice (KAP) model is a widely used behavioural framework in health promotion. ^12^ The present study is grounded in the KAP model, which posits that knowledge influences health attitudes, which in turn shape behavioural practices. Given the global trend toward westernized diets and the rapid nutrition transition in South and Southeast Asia, the findings from Puducherry reflect broader regional trends in adolescent nutrition.

 However, emerging evidence suggests that knowledge alone is often insufficient to produce behavioural change. Adolescents’ dietary choices are also mediated by environmental accessibility, peer influence, and socio-economic determinants. Therefore, the current study extends the traditional KAP framework by evaluating the direct and indirect behavioural pathways linking fast-food consumption and obesity outcomes using structural equation modeling (SEM) and receiver operating characteristic (ROC) curve analysis.^11^ This integrative theoretical approach allows for a comprehensive understanding of modifiable behavioural determinants relevant to obesity prevention and evaluates prevalence trends among the school-going adolescents.

## Methods

###  Study Design 

 The cross-sectional study was designed to analyze behavioural pathways, aiming to inform preventive strategies that target modifiable factors associated with adolescent obesity.

###  Participants

 This school-based study was conducted among students in the 11th and 12th standards in three government and private schools in urban Puducherry from January 2022 to June 2023.

###  Study Setting

 A multistage random sampling technique was used. The Villianur commune in Puducherry was selected purposively as the institution’s urban field practice area to ensure feasibility and administrative support. Three higher secondary schools were subsequently selected via random sampling. Within these schools, class sections from the 11th and 12th standards were listed, and students were selected using simple random sampling to ensure representation from both grades. All eligible students in the selected sections were enrolled until the required sample size was achieved. A sample size of 455 was calculated using the formula Z^2^ pq/d^2^ (95% confidence interval, 5% sampling error, and 10% non-response rate), based on a previously reported fast-food consumption prevalence of 36%. Students aged 15–18 years who provided informed assent for the study were included. Students who were absent for three consecutive visits at the time of the study were excluded.

###  Instrumentation

 Data were collected using a pre-designed, pre-tested, semi-structured questionnaire. The study protocol was reviewed by the Scientific Research Committee (SRC) and the study was conducted following approval from the Institutional Ethics Committee (IEC) and the relevant school authorities.

###  Measurement Validity

 The questionnaire was developed following an extensive literature review and expert consultation to ensure content validity. Public health and nutrition specialists reviewed the tool to assess its relevance, clarity, and cultural appropriateness. The construct validity of the behavioural framework was further examined using SEM, which confirmed significant theoretical relationships among KAP components, thereby supporting the measurement structure.

###  Operational Definitions

- Fast food/junk food: Foods that are commercially prepared, energy-dense, and nutrient-poor, including fried items, packaged snacks, sugar-sweetened beverages, baked confectionery, and ready-to-eat products high in fat, salt, or added sugars.
- Obesity and overweight: Nutritional status was assessed using BMI, calculated as weight (kg) divided by height (m^2^). Overweight and obesity were classified according to Asian BMI cut-off criteria (overweight: BMI ≥ 23.0 kg/m^2^; obesity: BMI ≥ 25.0 kg/m^2^).
- KAP scores: The scores related to fast food consumption were assessed using a structured questionnaire. Composite scores were calculated for each domain, with higher scores indicating better knowledge, more favourable attitudes, or healthier dietary practices.

###  Procedure

 The questionnaire was translated into the local language and back-translated to ensure linguistic consistency. It included sections on socio-demographic characteristics, fast-food consumption patterns, and KAP components related to fast food consumption. Socio-economic status was assessed using the modified B.G. Prasad classification (2020).

###  Scoring and Categorisation of Knowledge–Attitude–Practice

 KAP scores were categorized as “poor,” “ fair,” or “reasonable” based on tertile distribution derived from total scores. Thresholds were established based on questionnaire scoring and reflected adherence to recommended dietary behaviours and frequency of consumption, respectively. Anthropometric measurements were obtained using standard procedures. Weight was measured with a calibrated digital scale, and height was measured with a portable stadiometer. Trained investigators conducted all measurements to ensure reliability.

###  Data Analysis

 Statistical analysis was conducted using IBM SPSS for Windows (Armonk, NY: IBM Corp, v27.0.1). Chi-square tests were used to compare categorical associations. Pearson’s correlation was conducted to assess continuous variables. A chi-square test for linear trend was used to evaluate the dose–response relationship between the duration of fast-food consumption and obesity status. SEM was employed to examine the hypothesized relationships between knowledge, attitude, practice, and obesity. The multivariate technique allowed for the simultaneous evaluation of complex direct and indirect relationships between latent behavioural constructs and health outcomes. The model, constructed based on the theoretical KAP framework, hypothesized that knowledge influences attitude and practice, with practice mediating obesity outcomes. Estimation was performed using maximum likelihood in AMOS software. Model adequacy was evaluated using multiple fit indices, including the Comparative Fit Index (CFI), Root Mean Square Error of Approximation (RMSEA), Standardized Root Mean Square Residual (SRMR), and the chi-square to degrees of freedom ratio (χ^2^/df). ROC curve analysis was conducted to evaluate the discriminatory ability of the practice score in identifying adolescents with obesity.

 Finally, ROC curve analysis was conducted to evaluate the potential utility of behavioural practice scores as a practical tool for school health screening programmes rather than for diagnostic purposes. The area under the curve (AUC), sensitivity and specificity were calculated to assess the predictive performance.

###  Handling of Missing Data

 Incomplete questionnaires and records with missing anthropometric measurements were excluded. Only participants with complete data for knowledge, attitude, practice scores, and BMI were included in the final analysis, resulting in a final analytical sample of 430 participants.

## Results

 The mean (SD) age of study participants was 16.2 ± 0.7 years. The cohort consisted of approximately 81.2% females and 18.8% males. The prevalence of fast food/junk food consumption was 93.9%. Overall, 11.1% of participants were overweight, and 15.0% were obese. Most students reported consuming fast food primarily due to taste preference (85.1%), and 36.1% had been consuming fast food for more than five years.

 Participants were categorized based on their knowledge, attitude, and fast-food consumption practices, with corresponding scores assigned. Specifically, 79.3% of the participants had poor knowledge, 63.7% had a fair attitude, and 65.8% demonstrated good practices regarding fast-food consumption.A positive linear relationship was observed between knowledge score and participant age (r = 0.043).

 Chi-square analysis showed a significant association between participants’ fast-food and junk-food consumption practices and obesity (*P* = 0.016). A statistically significant association was also observed between age and knowledge scores (*P* = 0.030). Students aged 16–17 years had the highest proportion of poor knowledge (85%), followed by those aged 17–18 years (76.2%). Regarding practice scores, gender exhibited a statistically significant association (*P* = 0.046), with a greater proportion of females (68.6%) demonstrating good practices than males regarding fast food consumption (54.2%). In contrast, poor practice scores were relatively higher among males. Socio-economic status was also significantly associated with practice scores (*P* = 0.018), with better practices observed among students from higher socio-economic classes (classes I-IV), as depicted in Table 1.

**Table 1:** Association Between Socio-demographic Characteristics and KAP (N = 430)

**Characteristics**	**Good**		**Fair**		**Poor**		
	**Number**	**Percent**	**Number**	**Percent**	**Number**	**Percent**	***P*** **-value**
Knowledge Score							
Age Group (yr)							
15-16	3	4.4	17	25	48	70.6	0.030
16-17	10	5.2	19	9.8	165	85	
17-18	9	5.3	31	18.5	128	76.2	
Attitude Score							
Gender							
Male	11	13.3	43	51.8	29	34.9	0.001
Female	16	4.6	231	66.6	100	28.8	
Practice Score							
Gender							
Male	45	54.2	31	37.3	7	8.5	0.046
Female	238	68.6	90	25.9	19	5.5	
Socio-economic Status							
I	9	69.2	3	23.1	1	7.7	0.018
II	37	59.7	24	38.7	1	1.6	
III	104	72.2	31	21.5	9	6.3	
IV	102	69.4	35	23.8	10	6.8	
V	31	48.4	28	43.8	5	7.8	

*Note.* KAP, Knowledge, attitude, and practice.

 A significant association between practice score and obesity was observed among females (χ^2^ = 8.87, *P* = 0.012), indicating a higher prevalence of obesity among those with poor practices. However, no significant association was found among males (*P* = 0.384), as seen in Table 2. A significant linear trend was observed between increasing duration of fast-food consumption and obesity (χ^2^ = 6.74, *P* = 0.034), with obesity prevalence rising progressively with longer consumption duration (Table 3). This dose–response relationship supports a temporally and biologically plausible association between prolonged fast-food consumption and obesity.

**Table 2:** Association Between Practice Score and Obesity Status Stratified by Gender (N = 430)

**Characteristics**	**Good**		**Fair**		**Poor**		***P*** **-value**
	**Number**	**Percent**	**Number**	**Percent**	**Number**	**Percent**	
Females							
Obese	28	53.8	17	17	7	32.7	0.012
Non-obese	210	70.7	74	74	13	24.9	
Males							
Obese	7	53.8	3	3	3	23.1	0.384
Non-obese	38	55.9	27	27	3	39.7	

**Table 3:** Trend Between the Duration of Fast-Food Consumption and Obesity Status (N = 430)

**Duration of Fast-Food** **Consumption (yr)**	**Obese (n=650)**		**Non-obese (n=365)**		**Total**	**χ^2^ for trend**	***P*** **-value**
	**Number**	**Percent**	**Number**	**Percent**			
< 2	11	16.9	122	33.4	133	6.74	0.034
3–5	18	27.7	124	34.0	142		
> 5	36	55.4	119	32.6	155		

 SEM demonstrated that knowledge had a significant positive effect on attitude (β = 0.41, *P* < 0.001) and practice (β = 0.19, *P* = 0.045); attitude, in turn, influenced practice (β = 0.36, *P* < 0.001). practice was inversely associated with obesity (β = -0.28, *P* = 0.003). No direct effect of knowledge or attitude on obesity was observed. Model fit indices confirmed good model fit: CFI = 0.947, RMSEA = 0.048, SRMR = 0.032, χ^2^/df = 1.92. (Figure 1)

**Figure 1:**
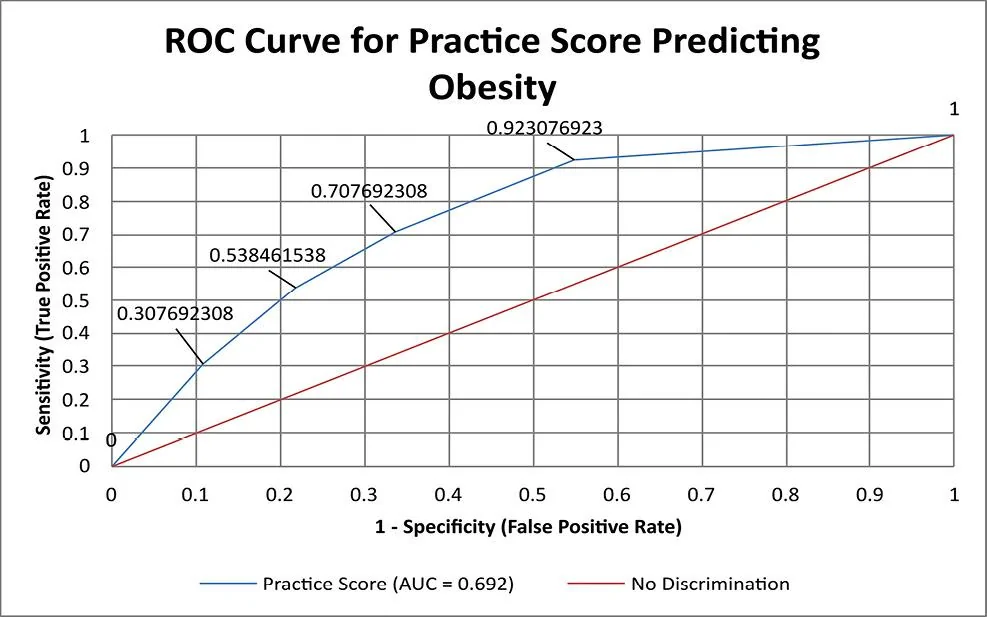
Structural Equation Modeling Structural equation model depicting the relationships among knowledge score, attitude score, and practice score in relation to adolescent obesity. Model Fit Indices: CFI = 0.947 (≥ 0.90) – very good fit; RMSEA = 0.048 (≤ 0.06) Excellent fit; SRMR = 0.032 (≤ 0.08). χ2/df = 1.92 (acceptable if < 3).
*Note.* SEM: Structural equation modeling; CFI: Comparative fit index; RMSEA: Root mean square error of approximation; SRMR: Standardized root mean square residual; df: degrees of freedom.

 ROC curve analysis indicated that the practice score moderately discriminated obesity status, yielding an AUC of 0.692 (95% CI: 0.625–0.759; *P* < 0.001). A cutoff value of ≤ 2.5 yielded a sensitivity of 70.8% and a specificity of 66.3%(Figure 2).

**Figure 2:**
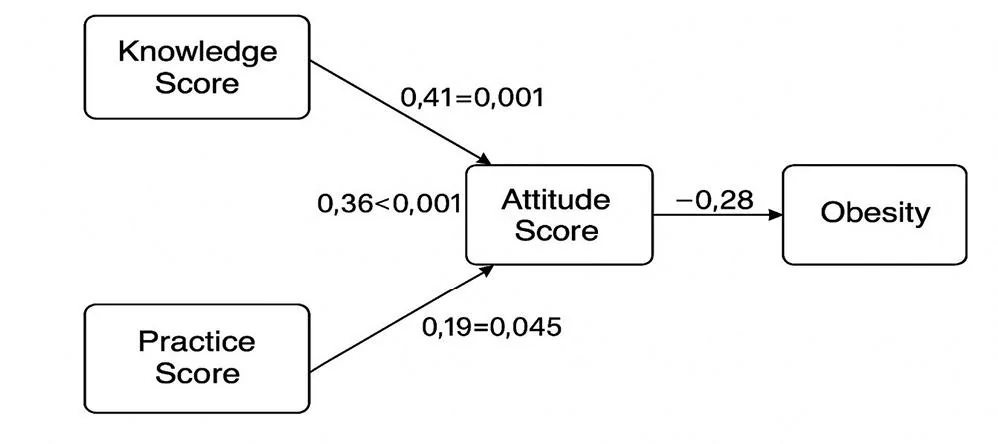
ROC Curve for Practice Score Predicting Obesity ROC curve showing the predictive ability of behavioral practice scores in identifying obesity. The AUC was 0.692 (95% CI: 0.625–0.759, *P* < 0.001), indicating fair discriminative power. The optimal cutoff value for the practice score was determined to be ≤ 2.5, yielding a sensitivity of 70.8% and a specificity of 66.3%.
*Note. *ROC: Receiver operating characteristic; AUC: Area under the curve; CI: Confidence interval.

## Discussion

 In this study, the majority of the adolescents consumed fast food/ junk food (93.9%), which is consistent with the findings of the survey conducted by Radhasri et al in Coimbatore.^15^ However, studies conducted in Delhi, Lucknow, Mangalore, Karnataka, Nepal, and Pune reported a lower prevalence than our observations, ranging from 47% to 80%. ^16,17,18^ These variations in prevalence may stem from differences in demographic profiles and dietary behaviors across regional settings.

 Factors such as taste preferences, ease of access, and lack of dietary regulation appear to drive unhealthy eating patterns, similar to findings reported by Sapkota and Neupane ^17^ in Chitwan and Majabadi et al in Tehran. ^19^ In contrast, studies by Ramesh Masthi and Jahan ^16^ in Bangalore and Kumari and Kumari ^20^ in Bihar observed that television advertisements for fast food/junk food, family socialization, changing lifestyles, and peer pressure were the primary factors influencing fast food/junk food habits among school-going adolescents.

 In our study, 79.3% of the participants had poor knowledge regarding fast food/junk food. Previous studies revealed that targeted nutrition awareness programs can significantly improve adolescent knowledge from 37% to 67% (*P* < 0.001). Although baseline knowledge was low in our study, the overall attitude toward fast food/junk food was fair (63.7%), which is consistent with the findings of Hu and Malik. ^21^ Furthermore, 65.8% of the students showed good practice on fast/junk food consumption. In contrast, a survey by Subedi et al in Nepal reported that 45.2% of participants had poor knowledge, and all participants consumed junk food. ^22^ The SEM findings validate the theoretical KAP model, confirming that students’ knowledge scores influence their practice indirectly through the mediating role of attitude. These findings align with previous studies among adolescents in Oman and India, emphasising the importance of behaviour-focused interventions over knowledge-focused strategies. ^23-24^

 Previous studies have shown that socioeconomic factors, the nutrition transition, online food purchasing behaviours, ultra-processed food consumption, and food marketing substantially influence adolescent dietary practices and obesity risk.^25-28^ From a behavioural standpoint, lower levels of physical activity and higher sedentary behaviour among adolescent girls may reduce metabolic compensation for unhealthy diets, making dietary practices a more dominant determinant of obesity risk in this group. ^29,30^ This interpretation is strongly supported by our SEM, which showed that practice had a direct effect on obesity, whereas knowledge and attitude influenced obesity only indirectly. The stronger practice–obesity association observed among females underscores the central mediating role of dietary behaviour identified in the SEM and suggests that interventions aimed at modifying food-related practices may yield greater benefits for female adolescents. Collectively, these findings emphasize the importance of gender-responsive, behaviour-focused strategies in adolescent obesity prevention.

 Additionally, the ROC analysis suggests that practice score could serve as a moderate predictor of obesity, potentially identifying at-risk students during routine school health screenings. This supports evidence from similar studies depicting gender-specific patterns among females, thereby strengthening the association between poor practice and obesity. ^5,12-15,19-20^ Overall, this study identifies practice behaviours as a critical modifiable determinant of adolescent obesity. Integrating structured dietary counselling sessions into school health programs and restricting access to energy-dense food outlets in school vicinities may yield measurable reductions in adolescent BMI.

 The observed behavioural pathways suggest that improving dietary practices is more critical than knowledge acquisition alone. The inverse association between adolescent practice scores and obesity highlights the potential value of practice-oriented, school-based interventions, including healthier food environments and behavioural change communication. Integrating simple practice-based dietary assessments within existing school health and non-communicable disease prevention frameworks may facilitate the early identification of at-risk adolescents and support timely preventive interventions.

 At the public health policy level, these findings support prevention-focused strategies targeting adolescent dietary practices. Policy actions should include enforcing healthy school canteen guidelines, regulating fast-food outlets near schools, and integrating routine dietary screening into school health services. Preventive strategies emphasizing behaviour-based nutrition education, peer-led interventions, and early risk identification may contribute to reducing adolescent obesity and long-term burden of non-communicable diseases. Integrating these insights into existing adolescent and school health programs could enhance their real-world impact through peer-led behaviour change communication.

## Limitations

- A self-administered questionnaire was used for data collection, which limits the ability to ascertain that the responses provided by the students were entirely genuine.
- Responses regarding dietary behaviours, such as the frequency of fast-food consumption over the past month, were subject to recall bias.
- The design precludes establishment of direct causal inference between fast-food consumption behaviours and obesity outcomes.

## Strengths

- The use of SEM allowed for assessing both direct and indirect behavioural pathways.
- The study utilized a large school-based adolescent sample and incorporated gender-stratified analysis.
- The integration of ROC curve analysis evaluates the practical screening of obesity risk.

**Highlights**

- Fast food consumption was highly prevalent (93.9%) among urban Indian adolescents, alongside a notable burden of adolescent obesity.
- Behavioural pathway analysis indicated that dietary practice was the strongest mediator linking knowledge and attitude to obesity outcomes.
- Practice score demonstrated moderate predictive ability for obesity; therefore, ROC analysis may be useful for school-based screening and prevention strategies.

## Conclusion

 Healthy eating is influenced by a variety of factors, including time, the availability of healthy options, friends’ eating habits, and nutritional knowledge. Despite the overall long-term health benefits of healthy eating, many students engage in poor dietary habits, such as high intake of fast food and other fatty foods, low consumption of fruits, vegetables, and dairy, and erratic eating habits, such as meal skipping. Practice plays a pivotal mediating role in obesity outcomes. Interventions targeting practical behaviour change rather than knowledge alone are likely to be more effective in preventing adolescent obesity. To conclude, the practice score could also serve as a screening tool for identifying students at risk.

## Acknowledgments

 The authors sincerely thank the school authorities, teachers, parents and students who participated in this study. The authors also acknowledge the support of the Department of Community Medicine, which facilitated data collection and provided technical guidance.

## Artificial Intelligence Use Disclosure

 Grammarly.com AI tool was used for only language editing and grammatical refinement. All AI-generated suggestions were reviewed, verified, and edited by the authors.

## Competing Interests

 There is no conflict of interests.

## Ethical Approval

 Ethical approval wasobtained from the Institutional Ethics Committee of SVMCH& RC (Ref No. 78/SVMCH/IEC/Jun 21).

## Funding

 This study received no specific grant.
